# Medicines and vaccines for the world's poorest: Is there any prospect for public-private cooperation?

**DOI:** 10.1186/1744-8603-1-10

**Published:** 2005-07-21

**Authors:** Richard M Scheffler, Vikram Pathania

**Affiliations:** 1 Director, The Nicholas C. Petris Center on Health Care Markets and Consumer Welfare, University of California, Berkeley, USA; 2 Distinguished Professor of Health Economics & Public Policy, University of California, Berkeley, USA; 3 University of California, Berkeley, 140 Warren Hall, MC7360 Berkeley, CA 94720-7360; 4 PhD Student, Department of Economics, University of California, Berkeley, USA

## Abstract

This paper reviews the current status of the global pharmaceutical industry and its research and development focus in the context of the health care needs of the developing world. It will consider the attempts to improve access to critical drugs and vaccines, and increase the research effort directed at key public health priorities in the developing world. In particular, it will consider prospects for public-private collaboration. The challenges and opportunities in such public-private partnerships will be discussed briefly along with a look at factors that may be key to success. Much of the focus is on HIV/AIDS where the debate on the optimal balance between intellectual property rights (IPR) and human rights to life and health has been very public and emotive.

## Introduction

Infectious diseases continue to place a great burden on the people in the developing world [1]. These diseases are for the most part controlled in developed countries. Since the global pharmaceutical industry is mostly grounded in developed countries, infectious diseases are not the prime focus of research and development (R&D). An important exception is HIV/AIDS therapy. This is a pressing matter for both developed and developing countries. But even in this case, the drug cocktails and disease management protocols are designed and priced to suit customers in the developed world. Pharmaceutical firms like all corporations aim to maximize shareholder value – their R&D and pricing decisions reflect this imperative. However, the needs and the paying capacity of the rich markets in the developed world are very different from those in the developing world. Consequently, the R&D agenda does not reflect most public health priorities in developing countries. Further, innovations in drugs and vaccines that are of potentially great benefit to developing countries are priced such that they are out of reach for most people in these countries [2]. Many find this situation deeply immoral and not in the best long-term interests of the world as a whole.

This paper reviews the current status of the global pharmaceutical industry and its R&D focus in the context of the health care needs of the developing world. It will consider the attempts to improve access to critical drugs and vaccines, and increase the research effort directed at key public health priorities in the developing world. In particular, it will consider prospects for public-private collaboration. The challenges and opportunities in such public-private partnerships will be discussed briefly along with a look at factors that may be key to success. Much of the focus is on HIV/AIDS where the debate on the optimal balance between intellectual property rights (IPR) and human rights to life and health has been very public and emotive.

## The Pharmaceutical Industry

**Figure 1:**
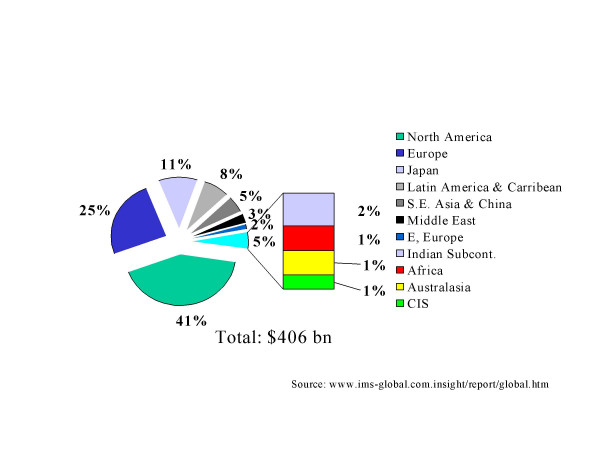
Global Pharmaceutical Market 2002.

The global pharmaceutical market was estimated at about US$406 billion in 2002. Figure 1 shows the geographical distribution. The US, Europe and Japan account for 77% of the market although they account for less than 15% of the global population [3]. In contrast, Sub-Saharan Africa that accounts for almost 25% of the global burden (measured in DALYs) of disease accounts for only 1% of the global spending.

Among the developed countries, the US dominates. It accounts for 38% of all global spending. The US market is huge and very important, and not just because it is the most populous of developed countries. Due to a relative absence of price controls, the unit realizations of pharmaceutical companies are higher in the US.

**Figure 2:**
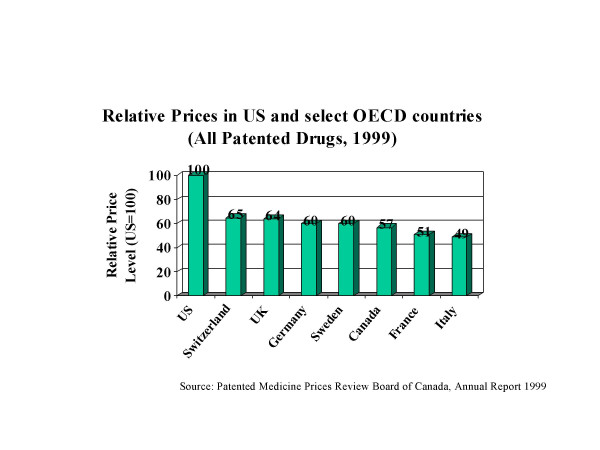
US is the Key Market.

Figure 2 shows the relative prices of a basket of all patented drugs in the US as compared to select OECD countries in 1999 [4]. Prices in the US are often twice as high. Thus, the US market is crucial important to the overall profitability of the industry, accounting for 60% of the global profits of the industry [5]. Therefore, the needs of the US market figure prominently in the priorities of decision makers in the industry and dictate much of the R&D agenda. In fact, until recently Europe has an edge in R&D spending and outcomes. Now the US has now come to dominate pharmaceutical R&D. In 2001, it spent over $30 billion on R&D as compared to $20 billion in Europe. In 1993–97, Europe launched 81 new molecular entities (NME) and America 48. But in 1998–2002, the respective figures were 44 and 85, almost an exact reversal [6]. In 2001, the major items on the R&D agenda were disorders linked to the Central Nervous System (26% of US R&D spending), Cancer, Endocrine & Metabolic Diseases (22%), and the cardiovascular system (18%.) Spending on research on developing an AIDS vaccine accounted for less than 1% of global R&D [7].

This skew in R&D focus is exacerbated by the nature of the R&D process. It is a long and uncertain road from the laboratory to the marketplace. Only 1 in 5000 promising molecules makes it to the product stage. On average, each new drug costs US$800 million in R&D costs [8]. It takes almost 12 years on average to get through all the stages of drug development. Most drugs do not contribute to profits; the industry depends on a handful of the so-called 'blockbuster' drugs. Examples of these drugs include the cholesterol lowering Lipitor by Pfizer and the anti-diabetic drug Glucophage by Bristols-Myers-Squibb. Blockbuster drugs have a rising share of the total market – from 6% in 1991 to 45% in 2001.

The rising uncertainty of payoffs from R&D is partly compensated by an increase in the effective patent life. Patent life has increased from about 8 years for drugs discovered in 1980 to as much as 13–15 years for recent discoveries [9]. Patent life is increased by: reducing the time spent in the approval process and testing, and extensions of patents. On the other hand, breakthrough drugs attract competition in form of slightly differentiated products even during the patent period; so pure monopoly is restricted effectively to 1–5 years. Post-patent, generics offer stiff competition and prices are marked down sharply.

**Figure 3:**
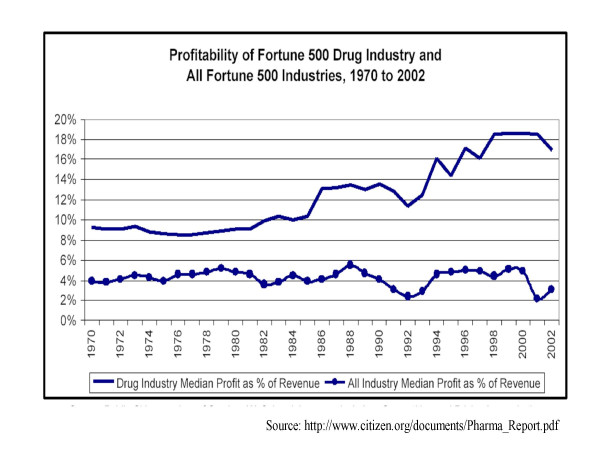
Profits in the Drug Industry.

In spite of the risky and expensive nature of the R&D process, the pharmaceutical industry as a whole is very profitable. Figure 3 shows that average profit margin for the industry from 1970 to 2002 [10]. It can be seen that the margin for the Fortune 500 drug firms has consistently exceeded the margin for all Fortune 500 companies taken together. It is possible that the higher profits are compensation for higher risk [11]. Also, pharmaceutical companies do not capitalize R&D costs. Instead, these are expensed as current costs. Therefore, successful drugs can generate high profits in accounting terms as the R&D costs for such drugs have already been expensed in the past.

Nevertheless, Table 1 shows that profits for the leading firms are well in excess of their current R&D outlays. The overall picture is one of an industry in robust health. This is a far cry from the picture often portrayed by industry advocates of an imperiled sector that would be pushed over the brink by price controls in developing countries.

**Table 1:** Top 5 by Drug Sales, 2002

**Company**	Pharma. Revenue* ($ bn)	**Net Profit ($ bn)**	**R&D ($ bn)**
Pfizer	28.29	9.13	5.18
GSK	27.06	6.96	4.11
Merck	20.13	7.15	2.67
AstraZeneca	17.84	2.84	3.07
Johnson & Johnson	17.15	6.6	3.96

*Most firms have income from other products as well; so total income exceeds pharmaceutical income

## Current Status in Access to Drugs

In this section, the current status in access to life saving drugs in developing countries is reviewed briefly. The focus is on access to HIV/AIDS drugs since the issue is topical, of great concern to major stakeholders, and illustrates the key issues. The World Health Organization estimates that 6 million AIDS patients can benefit immediately from Anti-Retroviral (ARV) therapy. However, only 300,000 are estimated to be currently accessing ARV [12]. This despite a sharp fall in the price of drugs in recent years. Table 2 shows the average price in US$ of ARV in May 2002 as compared to May 2001 in a few South American countries. Note that the price fell by as much as 85% in Haiti over this one-year period [13].

**Table 2:** Prices of Drugs in Selected Countries. Average Price in US$ of One year treatment with Anti Retro Viral (ARV: 3TC+AZT+EFV)

	**May 2001**	**May 2002**
Argentina	5182	1339
Brazil	1416	1307
El Salvador	6251	5583
Haiti	12569	1484
Mexico	3914	3820
Average	5506	2499

More recently, even lower prices have been quoted. Indian generic makers have quoted a price of $140 for a year's supply of generics for ARV while leading Western firms have quoted $500 per year for branded drug [14]. These prices are a small fraction of the market price in developed countries where ARV costs several thousand dollars a year. Table 3 shows the price difference between Brazil and Spain for commonly used anti-retroviral drugs in 2002 [15]. Brazilian prices are a fraction of the Spanish prices that often tend to be among the lowest in the OECD. Indian producers are offering generics at prices lower than those in Brazil. Clearly, branded and generics drugs are being offered at substantially lower prices in many developing countries.

**Table 3:** Price Variation in Select AIDS Drugs

**Drug**	**Unit**	**Brazil**	**Spain**
Aciclovir	250 mg (in vial)	1.25	4.87
Didanosine (ddl)	100 mg (tablet)	0.39	1.29
Efavirenz (EFZ)	200 mg (capsule)	0.85	3.3
Lamivudine (3TC)	150 mg (tablet)	0.29	2.7
Zidovudine (AZT)	100 mg (capsule)	0.13	0.79

***Indicative Ex-Work Prices in 2001, US$ ***Source: Sources and Prices of Selected Drugs and Diagnostics for People Living with HIV/AIDS, WHO/UNICEF/UNAIDS/MSF, 2003

Why are prices falling so fast? There are a number of reasons. First, there has been powerful advocacy by civil society, international development agencies, and many developing countries. While much of the push has been to lower prices on branded drugs and permit use of cheaper generics, there is also a growing effort to increase R&D in vaccines and drugs suited to needs in developing countries. Public-private partnerships to promote such research are an interesting new development and are covered in greater detail below. Second, there is active use of compulsory licensing by many developing countries. The World Trade Organization permits low-income countries to grant licenses for low-cost manufacture of patented drugs if these are deemed as essential to respond to serious public health threats. Third, there is also increasing use of parallel importation wherein countries import from the cheapest international source including generic manufacturers. Finally, countries have taken steps to reduce or eliminate import duties on drugs and pool procurement orders to gain bargaining leverage through higher volumes [16].

It should be noted though that even $140 per year is about half the per capita income in many African countries. Given the sheer scale of the epidemic, paying for treatment of all AIDS patients is beyond the capacity of these states; significant external funding is required to sustain such programs. One also has to add the investments and running costs required for upgrading and maintaining infrastructure to deliver AIDS care. Table 4 shows the relative cost-effectiveness of HIV interventions in the African setting. Clearly, prevention programs still deliver the best bang for the buck and should not be neglected in any AIDS control program [17]. Prevention should be a key priority for governments and donors.

**Table 4:** Estimates of the Cost-Effectiveness of HIV Interventions

	**Intervention in Africa, 2000**	**Cost per life year saved**
**Prevention**	Blood Screening	$3.35
	STD control & management for sex workers	$3.95
	Voluntary Counseling & Testing	$22.03
	Short-course ARV treatment for pregnant women (proposed)	$140*

**ARV treatment**	Generic Drugs (proposed)	$140*
	Patented Drugs	$560**
	Full price in 2000	$10707.09

*Clinton Foundation deal with Indian generic makers; **Price to Africa of HAART from big firms Source: Masaki E. et al. "Cost-effectiveness of HIV Interventions for Resource Poor Countries: Setting Priorities for HIV/AIDS management

## Industry Concerns: The Case for Property Rights

The brief discussion above shows that there is significant progress in reducing prices of ARV in developing countries. Pressure from advocacy groups and competition from cheap generics appear to have 'coerced' major pharmaceutical firms into marking down the prices of branded drugs in low income countries. But there are mounting industry concerns in this regard. Property Rights are considered the bedrock of a capitalist system and key to economic growth. Intellectual Property Rights (IPRs) are viewed as reward to innovation, and central to recouping R&D costs. Without such protection, firms warn that the incentive to invest in R&D is much attenuated with future generations around the world being the major losers [18].

The industry also has other major concerns. It fears that there will be legal or illegal re-importation of drugs into the rich markets given the huge price differential. This process can be seen unfolding in the fast growing volume of drug imports from Canada by US consumers. Further, the industry fears public pressure for price controls in key markets as consumers in developed countries become aware of low prices elsewhere. There is also fear of a domino effect: developing countries could demand lower prices for all patented drugs, not just for AIDS drugs. Finally, firms fear that without a deepening commitment to IPR, cheap generics will continue to dominate these potentially large and lucrative markets of the future.

The pharmaceutical firms' primary mission is to maximize shareholder value. They are partially justified in saying that they should not be made to pay for remedying the problem of inequitable access to drugs, a problem that is fundamentally driven by global economic inequity.

On the other hand, it can be argued that the right to life trumps the right to property. While the two rights are not mutually exclusive, they can be in immediate conflict as in the case of expensive life saving HIV/AIDS drugs and millions of impoverished AIDS patients around the world. Further, manufacturing and selling medicine is not quite the same as selling cars. Part of the mission of a pharmaceutical firm is to cure people. Also, exclusivity in the rights to ideas is questionable: new ideas build on existing knowledge. Often, private R&D uses freely available input in academic journals and conference proceedings; inputs that come from publicly funded universities and government laboratories. As a practical matter, continued foot dragging on the issue of global access to medicines may be poor corporate strategy. It generates adverse publicity, and animosity in developing countries that are destined to grow into the large markets of the future.

Thus far it seems that increasing access to drugs through lower prices has come largely through coercion of drug companies. However, there are recent systematic efforts to bring companies on board in public-private partnerships.

## Public-Private Partnerships

There are numerous partnerships that have sprung up in recent years. Among the notable ones are the Alliance for Microbicide Development, the Clinton Foundation HIV/AIDS Initiative, the Global Alliance for TB Drug Development, the International AIDS Vaccine Initiative, the Malaria Vaccine Initiative and the Medicines for Malaria Venture [19].

### Main Characteristics of the Partnerships

The partnerships share many structural characteristics [20]. They are usually constituted as independent legal entities. This aids in transparency and accountability. Further they may be viewed as relatively nonpartisan since they do not come encumbered with historical baggage. They have multiple partners from academia, industry, civil society, rich and poor countries, governments, and international agencies. The seed funding and some or much of the administrative costs is provided by public and philanthropic agencies. The pharmaceutical industry furnishes valuable in-kind contributions such as laboratory space, scientists' time, and access to databases. A key feature is that most of the partnerships recognize the basic validity of Intellectual Property Rights with some caveats. Indeed, this is crucial to gaining cooperation from the pharmaceutical companies.

### Keys to Successful Partnerships

Most partnerships are only a few years old and it is premature to pronounce a verdict on their effectiveness. However, even in this short time frame many have started to make major strides. Three select examples are discussed below. It is already possible to identify key success factors. Most partnerships share a mix of these factors although each may bring a unique proposition to the table to entice partners. First, many partnerships have charismatic leaders and spokespersons, e.g. the Clinton Foundation that is backed by former US president Bill Clinton and the icon of the anti-apartheid struggle, Nelson. Mandela. Second, the partnerships do not merely have strong advocacy skills but also display a keen business sense. An example is the business savvy Medicines for Malaria Venture (MMV.) This is very useful in dealing credibly with the large pharmaceutical firms. Third, the partnerships, perhaps by definition have to be adept at relationship management. They have to accommodate and influence the disparate agendas of multiple partners. For instance, the International AIDS Vaccine Initiative (IAVI) has 25 partners and operations in 22 countries. Fourth, partnerships often have technical expertise. They either employ clinical, epidemiological and laboratory experts or more likely have access to their services. Fifth, most partnerships have a sharp focus on one disease in their mission and operations. This in turn allows them to build up in-depth knowledge of the disease, epidemiological trends, the current status of R&D, and the market size and trends.

### The Clinton Foundation: Leveraging Charismatic Leadership

The foundation's HIV/AIDS Initiative is focused on supporting large-scale prevention & treatment in Caribbean & African countries. It develops country-level 'business' plans; and then presents these to donors and partners to mobilize resources. It has been very successful in reducing drug prices. It has been able to procure WHO-endorsed generics for ARV for as low as US$140 per year. Such low prices are now available to over 100 countries. In return, countries have to guarantee payment & secure drug distribution.

### Medicines for Malaria Venture: Demonstrating Business Savvy

Widespread drug resistance to older drugs has hampered malaria control in developing countries. Until recently, there was little R&D in new drugs or vaccines, as the disease had been all but eradicated in the developed world. MMV is a global public-private partnership of academia, government research groups, and pharmaceutical firms [21]. It develops and manages "virtual" R&D i.e. it does not own the physical infrastructure or employ many scientists but it leases these resources from companies. Clearly, this calls for considerable business skills. MMV aims for 1 new drug every 5 years at a cost of about US$150 million. This is significantly lower than the average cost of US$800 million for a new drug. The reduced costs are due to effective use of in-kind contributions from companies, simpler animal models and clinical trials, and pro bono governance and management.

### International AIDS Vaccine Initiative: Managing Relationships

The IAVI is focused on developing a vaccine to prevent HIV/AIDS in developing countries. It is involved in the entire gamut of operations to develop and test a vaccine – ranging from basic laboratory research to clinical tests. Its partners range from private laboratories to community-based organizations in developing countries that help recruit volunteers for clinical trials. In all, the IAVI has 25 partners and operations in 22 countries. It is the second largest supporter of AIDS vaccine R&D; it has committed US$100 million as of end-2003. The IAVI has five candidate vaccines under trial [22]. The IAVI retains the property rights to any future vaccine.

### What Is in it for Pharmaceutical Companies?

Why are pharmaceutical companies willing to participate in these partnerships? There are many reasons. First, in some cases they can retain the property rights to new medicines or vaccines developed. This is subject to their commitment to sell these products at marginal cost in developing countries. But they are free to make large profits in rich markets. Second. there are spin-off benefits from R&D. For instance, new knowledge gleaned from malaria R&D is potentially applicable in other products. Third, companies gain understanding of, and access to new markets. Fourth, smaller biotech firms can get into the spotlight, with higher visibility leading to more funding, and potentially big orders. Finally, companies can project themselves as good corporate citizens. Cooperation is usually a better option than legal confrontation and adverse publicity, and losing markets to generic manufacturers.

## Conclusion & Future Priorities

What are the major conclusions? It appears that the resource gap that is perceived as the main obstacle to access to drugs is shrinking. In part, this is due to an increased flow of resources from bilateral and multilateral agencies and private donors. But it is the rapidly falling price of drugs that has really helped reduce the resource gap. Providing ARV to millions of AIDS patients in developing countries at market prices is near impossible but becomes much more feasible at US$140 per year. Another key development is the emergence of focused public-private partnerships. The industry is being brought on board gradually. There are huge potential benefits if even a fraction of the industry's vast resources – laboratories, scientists, and databases can be harnessed to look for solutions to developing country health needs. As the partnerships strive to make the drug companies allies in the war on disease in developing countries, coordination across multiple partners will be a key challenge.

Coverage is still low – only a small fraction of patients are receiving drugs. Further, too little resources are devoted to R&D designed to address developing country needs that account for a huge portion of the global burden of disease. Advocacy groups should maintain pressure for lower prices. In this context, it is worth noting again that the industry is in robust financial health. The advocacy groups should also keep up pressure for increased funding from public and private donors. As the resource gap shrinks, strengthening public health infrastructure in developing countries will become a key priority. It is crucial to develop and put in place robust care delivery mechanisms that ensure smooth and secure flow of drugs, and maximize adherence to treatment protocols. Finally in the case of HIV/AIDS prevention should not be neglected. It is still the most cost-effective intervention, and therefore the most sustainable one in the long run.
